# Prevalence and Risk Factors of Anxiety, Depression, and Sleep Problems Among Caregivers of People Living With Neurocognitive Disorders During the COVID-19 Pandemic

**DOI:** 10.3389/fpsyt.2020.590343

**Published:** 2021-01-08

**Authors:** Qiuxuan Li, Haifeng Zhang, Ming Zhang, Tao Li, Wanxin Ma, Cuixia An, Yanmei Chen, Sha Liu, Weihong Kuang, Xin Yu, Huali Wang

**Affiliations:** ^1^National Health Commission Key Laboratory of Mental Health, National Clinical Research Center for Mental Disorders, Peking University Institute of Mental Health (Sixth Hospital), Beijing, China; ^2^Beijing Dementia Key Laboratory, Beijing, China; ^3^Department of Psychiatry, The Third Affiliated Hospital of Sun Yat-sen University, Guangzhou, China; ^4^Taiyanggong Community Health Center, Beijing, China; ^5^The First Hospital of Hebei Medical University, Shijiazhuang, China; ^6^The Third People's Hospital of Qinghai Province, Xining, China; ^7^Shanxi Key Laboratory of Artificial Intelligence Assisted Diagnosis and Treatment for Mental Disorders, First Hospital of Shanxi Medical University, Taiyuan, China; ^8^Department of Psychiatry, First Hospital/First Clinical Medical College of Shanxi Medical University, Taiyuan, China; ^9^Department of Psychiatry, Mental Health Center, West China Hospital, Sichuan University, Chengdu, China

**Keywords:** COVID-19, anxiety, depression, caregiver, neurocognitive disorders

## Abstract

**Objectives:** To estimate the prevalence of anxiety, depression, and sleep problems among caregivers of persons living with neurocognitive disorders (PLWND) during the COVID-19 pandemic in China and investigate whether the COVID-19-related experiences were associated with the presence of anxiety, depression, and sleep problems.

**Methods:** From March 1 to 31, 2020, 160 caregivers of PLWND participated in an online cross-sectional survey on the prevalence of anxiety, depression, and sleep problems. The 7-item Generalized Anxiety Disorder Scale (GAD-7) was administered to measure anxiety symptoms, and the 2-item Patient Health Questionnaire (PHQ-2) was used to assess depressive symptoms. Questions on sleep duration and sleep quality enquired about sleep problems. Six items were used to explore the COVID-19-related experiences, including community-level infection contact and the level of exposure to media information. We computed the prevalence rate of anxiety, depressive symptoms, and sleep problems. Univariate and multivariate logistic regression analyses were performed to investigate factors associated with these mental health problems.

**Results:** The prevalence rate of anxiety, depression, and sleep problems were 46.9%, 36.3%, and 9.4%. Approximately 55 participants (34.4%) presented with two or more mental health problems. Women had a higher risk of developing anxiety symptoms (OR, 5.284; 95% CI, 2.068–13.503; *p* = 0.001). Having a mental disorder (OR, 5.104; 95% CI, 1.522–17.114; *p* = 0.008) was associated with an increased risk of depressive symptoms. Caregivers who preferred to access positive information (OR, 0.215; 95% CI, 0.058–0.793; *p* = 0.021) was associated with decreased risk of sleep problems.

**Conclusion:** Anxiety and depressive symptoms were common among caregivers of older adults with dementia or mild cognitive impairment during the COVID-19 pandemic. Being female was an independent risk factor for experiencing anxiety symptoms. Preexisting mental disorders increased the risk of depressive symptoms among caregivers, while caregivers who prefer to access positive media information decreased sleep problems.

## Introduction

Family caregivers of persons living with neurocognitive disorders (PLWND), including dementia or mild cognitive impairment (MCI), often describe the experience as “enduring stress and frustration” (1). More than 80% of PLWND reported stressful experiences, including physical strains, psychological responses, and social isolation (2, 3). Previous studies observed that mental health problems such as anxiety, depression, and sleep problems were prominent among caregivers, especially those taking care of people with challenging behaviors (4).

During the COVID-19 pandemic, PLWNDs may become stressed when exposed to a replacement of caregivers, e.g., the children, instead of the domestic helper (5). Older adults living with dementia were vulnerable during the COVID-19 outbreak because they had limited access to accurate information, difficulty in remembering safeguard procedures, forgetting the warnings, and lacking sufficient self-quarantine measures. The COVID-19 pandemic may expose PLWND to a higher risk of infection and increased caregivers' concerns (6). The double hit of COVID-19 pandemics and the mental burden of caring for PLWND brought about significant problems (7).

The caregivers of PLWND tended to experience mental stress and feel isolated and helpless (8). Previous studies have shown that caregivers suffer from anxiety, fatigue, sleep disorders, and other mental health problems when in close contact with patients with emerging infectious diseases such as SARS (9), MERS-CoV infection (10, 11), Ebola (12), and H1N1 infection (13). During the COVID-19 outbreak, PLWNDs were likely to develop challenging behaviors, leading to a more significant caregiver burden in both physical and psychological aspects (7). For example, caregivers not only had higher workloads of caring for the PLWNDs but also worried about the PLWNDs' physical conditions in case of virus infection. However, the existing studies scarcely estimated the prevalence of anxiety, depressive symptoms, and sleep problems among caregivers of PLWND in China.

Besides, the associated factors related to these mental health problems were not studied well during the COVID-19 pandemic. The social-distancing measures implemented during the challenging time may impose additional pressure on the caregivers. Previous studies found that caregivers' support groups may benefit caregivers in reducing their stress (14). During the pandemic, the face-to-face caregiver support service was suspended and later transited to virtual meetings. In case of any suspected or confirmed cases of COVID-19 in the community, strict lockdown regulation would be implemented locally. The social network and connectedness among caregivers and service providers may be compromised. Also, when lived in such an isolated environment, family members may worry about the unknown conditions of severe emergencies and the protective equipment's short supply (7, 15). Whether the COVID-19 related experience affected the mental health status of caregivers remained unclear.

Previous studies have also shown that sensationalized media reports disseminated unauthorized information might even cause public panic (16–18). During the COVID-19, TV news, internet websites, and social media such as WeChat and WeBlog became mainstream information exchange and communication. Information related to COVID-19 flooded in daily life. Gao et al. found that a high prevalence of mental health problems was associated with frequent social media exposure during the COVID-19 outbreak (19). One explanation was that misinformation might drive fear, anxiety, and worries during unprecedented times. However, the impact of media exposure on the mental health status of the caregivers of PLWND was not studied. Additionally, whether the type of messages and the channels of communications were associated with the prevalence of mental health problems has yet to be elucidated.

Therefore, we hypothesized that mental health problems, particularly anxiety, depression, and sleep problems, were common among caregivers of PLWND. The high prevalence of these symptoms was also associated with the community-level COVID-19 contact and the level, nature, and channels of media exposure. To test these hypotheses, we conducted a cross-sectional survey during the COVID-19 outbreak. The study primarily aimed to estimate the prevalence of anxiety symptoms, depressive symptoms, sleep problems, and the coexistence of these three problems during the COVID-19 pandemic in China. Secondly, it aimed to explore whether the COVID-19-related experiences were associated with the risk of these mental health problems.

## Methods

From March 1 to 31, 2020, the cross-sectional survey was conducted among older adults' family caregivers through an anonymous online questionnaire through the Questionnaire Star platform. The URL link was distributed through the geriatric mental health service network by members of the Chinese Society of Geriatric Psychiatry. The online survey was first disseminated to older adults' caregivers and encouraged them to pass it on to other caregivers. To improve the response rate, the questions about mental health status were set as required items. The respondents would receive a reminder if some questions were missed during the survey. For the present study analysis, we identified the family caregivers who took care of persons with dementia or mild cognitive impairment as the study participants.

### Study Participants

**Figure 1:**
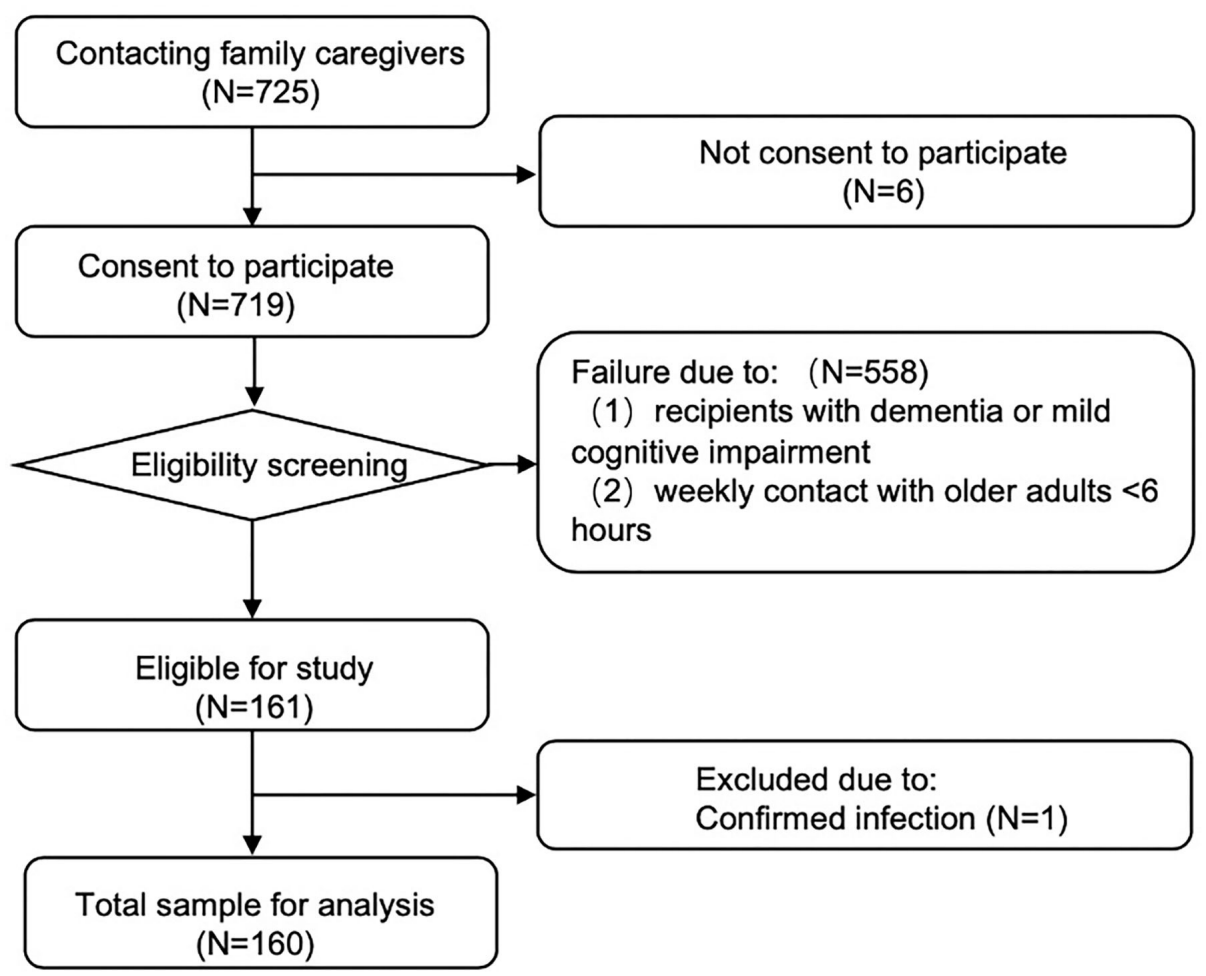
Flow chart of subject recruitment and participation in the online survey.

As illustrated in Figure 1, 160 caregivers of PLWND participated in the study. The inclusion criteria for caregivers of PLWND included family members who (1) took care of PLWND at home and (2) spent at least 6 h per week with care recipients. All caregivers must have essential listening, speaking, reading, and writing abilities and could use a smartphone or computer to ensure their completion of the self-reported questionnaire and online surveys.

The ethics committee of Peking University Sixth Hospital approved the study protocol. All subjects provided their consent by answering the screening question, “Are you willing to participate in the survey?” The survey was anonymous. No personal information could be identified in the questionnaire.

### Screening of Mental Health Problems

The 7-item Generalized Anxiety Disorder Scale (GAD-7) was used to screen for anxiety symptoms. The cutoff score for anxiety was ≥5. The two-item Patient Health Questionnaire (PHQ-2) inquiring of loss of interest and the low mood was used to screen for depressive symptoms. The cutoff score for depression was ≥2 (20). Two questions were used to screen for sleep problems: “How long on average did you sleep per day in the past month?” and “How has your sleep quality changed in the past month?” Sleep problems were defined as follows: (1) a daily average duration of sleep < 4 h or > 8 h, and (2) more reduced sleep quality than before.

### Evaluation of the COVID-19-Related Experiences

Six questions were used to evaluate the COVID-19-related experiences, including two items on the community-level infection contact and four questions on the level of exposure to media information (see Appendix in the Supplementary Document).

The questions examining the community-level infection contact were: “Did you have close contact with any individual with confirmed or suspected COVID-19?” and “Was there anyone confirmed or suspected with COVID-19 in your community and neighborhood?.” A response of “yes” to either question indicated a positive experience of community-level infection contact.

Four questions were used to measure the level of exposure to media information: the time spent browsing information per day (<1, 1–3, 3–6, or >6 h); the individual's preference to access the nature of media information (primarily positive, half positive/half negative or mostly negative), and the “positive” information means bring support and hope, the “negative” information means fear and panic; the number of channels used to obtain information (including TV news, the internet, social media platforms such as WeChat and WeBlog, the newspaper, relatives and friends, community workers, or others); and the reliability of the information obtained (information from TV, newspaper and community workers was classified as highly reliable; information from other channels was classified as potentially reliable).

### Medical History

Two questions identified the medical history of physical and mental conditions: “Have you ever been diagnosed with any of the following physical diseases, including hypertension, diabetes, heart disease, chronic bronchitis, stroke, Parkinson's disease, chronic renal insufficiency, chronic pain, or others?” and “Have you ever been diagnosed with any of the following mental disorders, including depressive disorders, anxiety disorders, obsessive-compulsive disorders, schizophrenia, dementia, mild cognitive impairment, or others?”

### Statistical Analysis

Data analysis was performed using SPSS statistical software version 26.0. The significance level was set at *p* < 0.05.

The prevalence of depressive symptoms, anxiety symptoms, and sleep problems was calculated using the cutoff mentioned above scores and reported as the percentages of cases in different populations. Participants were classified as a normal comparison, having any single mental health problem, or having multiple mental health problems. χ^2^ tests were used to compare the subjects' demographic characteristics, including age, sex, education level, marital status, place of residence, medical history, degree of community-level infection contact, and level of exposure to pandemic information between subgroups. We also performed Chi-Square tests to compare the demographic characteristics, COVID-19 related experiences, and mental health status between caregivers of people with dementia and MCI.

To explore the potential associated factors with anxiety, depressive symptoms, and sleep problem, we performed multiple logistic regression analyses and calculated odds ratios (ORs) and 95% CIs. The covariates included sex, history of preexisting mental disorders, degree of community-level infection contact, and level of exposure to pandemic information. As there was no significant difference in age, educational level, history of physical conditions, marital status, place of residence, we did not include these variables as covariates in the logistic regression analysis.

## Results

As presented in Table 1, approximately three-quarters of caregivers were women, and most participants were younger than 60 years old, married, and residing in cities. About 37.5% of the caregivers had physical conditions, and 8.8% had preexisting mental disorders.

**Table 1:** Comparisons of demographic characteristics and COVID-19-related experiences among mental health status subgroups.

**Variable**	**All home caregivers (*N* = 160)**	**Anxiety**		***p-*value**	**Depression**		***p-*value**	**Sleep problems**		***p-*value**
		**No (*N* = 85)**	**Yes (*N* = 75)**		**No (*N* = 102)**	**Yes (*N* = 58)**		**No (*N* = 145)**	**Yes (*N* = 15)**	
**Age**										
<60 years	112 (70.0%)	59 (52.7%)	53 (47.3%)	0.863	69 (61.6%)	43 (38.4%)	0.389	102 (91.1%)	10 (8.9%)	0.767
≥60 years	48 (30.0%)	26 (54.2%)	22 (45.8%)		33 (68.8%)	15 (31.3%)		43 (89.6%)	5 (10.4%)	
**Gender**										
Men	35 (21.9%)	28 (80.0%)	7 (20.0%)	<0.001	25 (71.4%)	10 (28.6%)	0.285	30 (85.7%)	5 (14.3%)	0.259
Women	125 (78.1%)	57 (45.6%)	68 (54.4%)		77 (61.6%)	48 (38.4%)		115 (92.0%)	10 (8.0%)	
**Schooling educational level**										
≤9 years	34 (21.3%)	16 (47.1%)	18 (52.9%)	0.424	22 (64.7%)	12 (35.3%)	0.896	32 (94.1%)	2 (5.9%)	0.431
>9 years	126 (78.8%)	69 (54.8%)	57 (45.2%)		80 (63.5%)	46 (36.5%)		113 (89.7%)	13 (10.3%)	
**Marital status**										
Married	137 (85.6%)	73 (53.3%)	64 (46.7%)	0.921	88 (64.2%)	49 (35.8%)	0.756	123 (89.8%)	14 (10.2%)	0.371
Single/divorced/widowed	23 (14.4%)	12 (52.2%)	11 (47.8%)		14 (60.9%)	9 (39.1%)		22 (95.7%)	1 (4.3%)	
**Residence**										
Urban	144 (90.0%)	74 (51.4%)	70 (48.6%)	0.187	92 (63.9%)	52 (36.1%)	0.913	130 (90.3%)	14 (9.7%)	0.651
Suburban/rural	16 (10.0%)	11 (68.8%)	5 (31.3%)		10 (62.5%)	6 (37.5%)		15 (93.8%)	1 (6.3%)	
**Physical conditions**										
Yes	60 (37.5%)	25 (41.7%)	35 (58.3%)	0.024	37 (61.7%)	23 (38.3%)	0.671	53 (88.3%)	7 (11.7%)	0.441
No	100 (62.5%)	60 (60.0%)	40 (40.0%)		65 (65.0%)	35 (35.0%)		92 (92.0%)	8 (8.0%)	
**Preexisting mental disorders**										
Yes	14 (8.8%)	3 (21.4%)	11 (78.6%)	0.013	4 (28.6%)	10 (71.4%)	0.004	11 (78.6%)	4 (21.4%)	0.105
No	146 (91.3%)	82 (56.2%)	64 (43.8%)		98 (67.1%)	48 (32.9%)		134 (91.8%)	12 (8.2%)	
**Community-level infection contact**										
Yes	33 (20.6%)	15 (45.5%)	18 (54.5%)	0.322	18 (54.5%)	15 (45.5%)	0.217	30 (90.9%)	3 (9.1%)	0.950
No	127 (79.4%)	70 (55.1%)	57 (44.9%)		84 (66.1%)	43 (33.9%)		115 (90.6%)	12 (9.4%)	
**Time spent browsing information**										
<1 h	35 (21.9%)	19 (54.3%)	16 (45.7%)	0.721	20 (57.1%)	15 (42.9%)	0.789	30 (85.7%)	5 (14.3%)	0.156
1-3 h	87 (54.4%)	43 (49.4%)	44 (50.6%)		56 (64.4%)	31 (35.6%)		79 (90.8%)	8 (9.2%)	
3-6 h	28 (17.5%)	17 (60.7%)	11 (39.3%)		19 (67.9%)	9 (32.1%)		28 (100%)	0 (0%)	
>6 h	10 (6.2%)	6 (60.0%)	4 (40.0%)		7 (70.0%)	3 (30.0%)		8 (80.0%)	2 (20.0%)	
**Preference for the nature of information**										
Primarily positive	81 (50.6%)	47 (58.0%)	34 (42.0%)	0.209	54 (66.7%)	27 (33.3%)	0.437	78 (96.3%)	3 (3.7%)	0.013
Half positive/half negative or primarily negative	79 (49.4%)	38 (48.1%)	41 (51.9%)		48 (60.8%)	31 (39.2%)		67 (84.8%)	12 (15.2%)	
Number of channels used to obtain information		2.82 ± 1.255	3.05 ± 1.283	0.836	2.86 ± 1.227	3.05 ± 1.343	0.372	2.90 ± 1.284	3.27 ± 1.100	0.470
Reliability of the information obtained		2.04 ± 0.933	2.12 ± 0.982	0.951	2.06 ± 0.930	2.10 ± 1.003	0.650	2.07 ± 0.967	2.20 ± 0.841	0.275

Of all study participants, 75 (46.9%) presented with significant anxiety symptoms, 58 (36.3%) had depression symptoms, and 15 (9.4%) reported sleep problems. As shown in Tables 1, 2, anxiety symptoms were more frequent among women than men (54.4 vs. 20.0%, χ^2^ = 12.994, *p* < 0.001), among caregivers with physical conditions than those who were healthy (58.3 vs. 40.0%, χ^2^ = 5.061, *p* = 0.024), and among caregivers with preexisting mental disorders than those without (78.6 vs. 43.8%, χ^2^ = 6.190, *p* = 0.013). Depression symptoms were more common among caregivers with preexisting mental conditions than healthy (71.4 vs. 32.9%, χ^2^ = 8.216, *p* = 0.004). Caregivers who were preferred to access positive information than obtain half positive/half negative or primarily pessimistic information had a lower prevalence of sleep disturbance (3.7 vs. 15.2%, χ^2^ = 6.210, *p* = 0.013). There were no significant differences in demographic characteristics, COVID-19-related experiences (see Supplementary Table 1), the prevalence of anxiety, depressive symptoms, and sleep problems (see Supplementary Table 2) between people's caregivers with dementia and MCI.

**Table 2:** Univariate and multivariate analysis of factors associated with anxiety, depression, and sleep problems.

	**Univariate analysis**						**Multivariate analysis**					
**Variables**	**GAD-7**		**PHQ-2**		**Sleep problem**		**GAD-7**		**PHQ-2**		**Sleep problem**	
	**χ^2^**	***p*-value**	**χ^2^**	***p*-value**	**χ^2^**	***P*-value**	**OR (95% CI)**	***p*-value**	**OR (95% CI)**	***p*-value**	**OR (95% CI)**	***p*-value**
Age	0.030	0.863	0.742	0.389	0.088	0.767						
Gender	12.994	<0.001	1.143	0.285	1.272	0.259	5.284 (2.068–13.503)	0.001				
Schooling education level	0.638	0.424	0.017	0.896	0.620	0.431						
Marital status	0.010	0.921	0.096	0.756	0.799	0.371						
Place of residence	1.743	0.187	0.012	0.913	0.204	0.651						
Physical conditions	5.061	0.024	0.180	0.671	0.593	0.441	2.011 (0.960–4.216)	0.064				
Preexisting mental disorders	6.190	0.013	8.216	0.004	2.624	0.105	3.099 (0.732–13.217)	0.125	5.104 (1.522–17.114)	0.008		
Community-level infection-contacting experience	0.982	0.322	1.524	0.217	0.004	0.950						
Time spent on reading media messages	1.335	0.721	1.049	0.789	5.222	0.156						
Preference to the media messages	1.582	0.209	0.604	0.437	6.210	0.013					0.215 (0.058–0.793)	0.021
Number of channels to obtain relevant information	0.043	0.836	0.803	0.372	0.525	0.470						
Reliability of obtained information	0.004	0.951	0.206	0.650	1.200	0.275						

Multivariate regression analysis showed that female sex (OR = 5.284, 95% CI = 2.068–13.503, *p* = 0.001) was associated with an increased risk of experiencing anxiety symptoms. Preexisting mental disorders (OR = 5.104, 95% CI = 1.522–17.114, *p* = 0.008) were associated with an increased risk of depression symptoms. Preferring access to positive messages (OR = 0.215, 95% CI = 0.058–0.793, *p* = 0.021) may reduce sleep problems (Table 2).

Among all participants, 55 (34.4%) had two or more mental health problems, of which ~80% of the subjects had anxiety symptoms and depressive symptoms simultaneously. As shown in Table 3, having two or more types of symptoms was more prevalent among caregivers who had preexisting mental disorders than those without (71.4 vs. 30.8%, χ^2^ = 11.123, *p* = 0.004). However, the effect of preexisting mental conditions on multimorbidity was not significant in the multivariate analysis.

**Table 3:** Comparisons of demographic characteristics and COVID-19-related experiences among those with mental health multimorbidity, those with single morbidity, and normal controls.

**Variable**	**Normal controls (*N* = 73)**	**Single morbidity (*N* = 32)**	**Multimorbidity (*N* = 55)**	**χ^2^**	***p*-value**
**Age**					
<60 years	50 (44.6%)	22 (19.6%)	40 (35.7%)	0.298	0.862
≥60 years	23 (47.9%)	10 (20.8%)	15 (31.3%)		
**Gender**					
Women	52 (41.6%)	25 (20.0%)	48 (38.4%)	4.722	0.094
Men	21 (60.0%)	7 (20.0%)	7 (20.0%)		
**Schooling educational level**					
≤9 years	13 (38.2%)	10 (29.4%)	11 (32.4%)	2.480	0.289
>9 years	60 (47.6%)	22 (17.5%)	44 (34.9%)		
**Marital status**					
Married	62 (45.3%)	28 (20.4%)	47 (34.3%)	0.121	0.941
Single/divorced/widowed	11 (47.8%)	4 (17.4%)	8 (34.8%)		
**Residence**					
Urban	65 (45.1%)	28 (19.4%)	51 (35.4%)	0.751	0.687
Suburban/rural	8 (50.0%)	4 (25.0%)	4 (25.0%)		
**Physical conditions**					
Yes	21 (35.0%)	15 (25.0%)	24 (40.0%)	4.459	0.108
No	52 (52.0%)	17 (17.0%)	31 (31.0%)		
**Preexisting mental disorders**					
Yes	1 (7.1%)	3 (21.4%)	10 (71.4%)	11.123	0.004
No	72 (49.3%)	29 (19.9%)	45 (30.8%)		
**Community-level infection contact**					
Yes	13 (39.4%)	5 (15.2%)	15 (45.5%)	2.327	0.312
No	60 (47.2%)	27 (21.3%)	40 (31.5%)		
**Time spent browsing information**					
<1 h	14 (40.0%)	7 (20.0%)	14 (40.0%)	4.842	0.564
1–3 h	39 (44.8%)	17 (19.5%)	31 (35.6%)		
3–6 h	16 (57.1%)	4 (14.3%)	8 (28.6%)		
>6 h	4 (40.0%)	4 (40.0%)	2 (20.0%)		
**Preference for the nature of information**					
Primarily positive	42 (51.9%)	15 (18.5%)	24 (29.6%)	2.649	0.266
Half positive/half negative or primarily negative	31 (39.2%)	17 (21.5%)	31 (39.2%)		
Number of channels used to obtain information	2.79 ± 1.269	3.09 ± 1.201	3.02 ± 1.312	0.634	0.674
Reliability of the information obtained	2.03 ± 0.951	2.19 ± 0.914	2.07 ± 0.993	0.569	0.802

## Discussion

Our study found that approximately half of the caregivers of old adults with neurocognitive disorders had anxiety symptoms, and two-fifths of caregivers had depression symptoms during the COVID-19. The study also identified that females had a higher risk of anxiety symptoms; those having preexisting mental disorders were more likely to develop depression symptoms, while enhanced access to positive media information decreased the risk of sleep problems.

Our study found that anxiety and depressive symptoms were common among caregivers of PLWND. The estimates were higher than those reported during routine care before the COVID-19 outbreak (21). As we have observed, during this COVID-19 pandemic, family members who took care of persons with dementia were exposed to physical and psychological stress, which may contribute to a more significant caregiver burden (5). A study previously found that family caregivers reported substantial emotional and social burdens even within the 1st year of receiving a diagnosis of Alzheimer's disease. The caregiver burden increased with the concerns of behavioral problems (22). During the COVID-19 outbreak, older adults with dementia relied on their caregivers to manage household chores due to the restriction of mass transportation and outdoor activities. Thus, the physical and time burden was increased. In addition to being concerned about the risky situation of the contagious disease, the caregivers may become exhausted with a feeling of suffering and burnout (23).

The caregiver gender was frequently reported to be associated with caregivers' mental health problems. Our study observed that female caregivers had a higher risk of anxiety symptoms. The finding was consistent with previous studies (24). Globally, almost 80% of the caregivers are women as they could be the wife, daughter, or daughter-in-law of the person with dementia (25). Caring for dementia predicted a higher level of burden. Gender might influence the individual's kinship roles and personal perceptions of caregiving burden. Previous studies have found that female caregivers tended to report more mental health problems than male caregivers (26, 27). The help-seeking behaviors of the caregivers might account for such a tendency. Almberg et al. found gender differences in coping with the caregiving burden: men expressed a need for social support; women showed a positive attitude toward the relationships with other family members and thus exhibited more mental health problems. Men might have more access to external resources for help, which alleviated the stress and burden (26).

Our survey showed that caregivers who had preexisting mental disorders were at a higher risk of depression symptoms during the COVID-19. Patients with severe mental illness may be the most vulnerable populations when facing disaster (28). Those caregivers who have severe mental illnesses may have difficulty taking time off from the care recipient and may lack sufficient insurance to cover testing and treatment (29). The caregivers with preexisting mental health problems in our study also had a high tendency to present several symptoms. The mental health problems of the caregivers were overlooked, especially during the social distancing period. Caregivers who had poor psychological health, high depressive symptoms, and elevated anxiety symptoms experienced a more significant burden from their caregiving (4). The greater burden may lead to job dissatisfaction and possibly further impair work performance and aggravate more burnout experiences. Such problems were prominent during the outbreak of COVID-19 (29). These findings highlight that timely and continuously mental health care needs to be developed urgently. However, further studies are warranted to explore which preexisting mental disorders were more specific to trigger depressive symptoms during the COVID-19 pandemic and other emergencies.

Interestingly, our study revealed that caregivers who preferred to obtain favorable media information decreased the risk of sleep problems. During the COVID-19, one of the most stressful situations was the contagion's unpredictability and uncertainty. These, along with misinformation, might raise concerns in public. An overabundance of misinformation on social media imposed a significant risk to public mental health during the pandemic crisis (19, 30). Preference for positive media information would allow an individual to consider the potential risk and challenges critically. Timely access and utilization of accurate information may benefit the mental well-being of caregivers.

## Limitations

The research findings need to be interpreted with caution, as there are several limitations. First, the subject selection bias was innegligible in the online survey. Only those willing to discuss psychology-related topics were counted, while those who refused were not investigated thoroughly. Second, during the study period, it was not feasible to reach individual caregivers due to social distancing regulations. The online survey may not be universally accessible by all relevant stakeholders of caregivers. For example, spouse caregivers might not participate because they might have limited access to mobile technology. Therefore, it would be more informative to conduct face-to-face or telephone-based interviews with spouse caregivers in future studies. Last, it remained controversial whether the inclusion of caregivers of individuals with MCI would lower the estimates of the prevalence of mental stress. One might argue that old adults with MCI by definition preserve autonomy in everyday life, implying that their caregivers' role could be quite different from that in dementia. In fact, during the COVID-19 outbreak, memory problems in MCI affected family caregivers more often than usual. For example, repeated asking questions interfered with the caregivers who worked at home during the pandemic. In our study, there was no difference in the mental health status between subgroups of dementia and MCI. However, the distribution of the two subgroups was uneven and prevented from the subgroup analysis. Therefore, we advise that the caregivers' roles and psychological responses in a different cognitive impairment stage need further investigations.

## Conclusion

Anxiety and depression were prevalent among caregivers of neurocognitive disorders during the COVID-19 epidemic, especially among females and those with preexisting mental conditions. The findings highlight that, during unprecedented times, continuous mental health care is warranted for caregivers of persons living with neurocognitive disorders. However, the mental health status of caregivers should be monitored during the post-epidemic era.

## Data Availability Statement

The datasets generated and analyzed during the current study are not publicly available because we are preparing an additional manuscript. However, they are available upon reasonable request to the corresponding author, HW (huali_wang@bjmu.edu.cn).

## Ethics Statement

The studies involving human participants were reviewed and approved by Peking University Sixth Hospital. Written informed consent for participation was not required for this study in accordance with the national legislation and the institutional requirements.

## Author Contributions

QL and HZ contributed to the study design, data collection, analysis, interpretation, and drafted the manuscript. MZ, TL, and WM contributed to the study design, data collection, analysis, and interpretation. CA, YC, SL, and WK contributed to the data collection, research, and interpretation. XY and HW conceived the study, contributed to data interpretation, and critical revision of the manuscript. HW had primary responsibility for the final content. All authors read and approved the final manuscript.

## Conflict of Interest

The authors declare that the research was conducted in the absence of any commercial or financial relationships that could be construed as a potential conflict of interest.
