# Supplementary material for: Prevalence and Risk Factors of Anxiety, Depression, and Sleep Problems Among Caregivers of People Living With Neurocognitive Disorders During the COVID-19 Pandemic
Source: Front Psychiatry. 2021 Jan 8;11:590343. doi: 10.3389/fpsyt.2020.590343 (PMC7820074; doi:10.3389/fpsyt.2020.590343)
Supplement: Supplementary file 1 [file Data_Sheet_1.PDF]

Table S1. Comparisons of demographic characteristics and COVID-19-related experiences between caregivers of persons living with dementia and mild cognitive impairment

Variable	Dementia No. (%)	MCI No. (%)	χ^2	*p*-value
Age				
<60 years	69 (68.3%)	27 (71.1%)	0.097	0.756
≥60 years	32 (31.7%)	11 (28.9%)		
Gender				
Women	79 (78.2%)	29 (76.3%)	0.058	0.810
Men	22 (21.8%)	9 (23.7%)		
Schooling educational level				
≤9 years	16 (15.8%)	11 (28.9%)	3.030	0.082
>9 years	85 (84.2%)	27 (71.1%)		
Marital status				
Married	86 (85.1%)	35 (92.1%)	1.185	0.276
Single/divorced/widowed	15 (14.9%)	3 (7.9%)		
Residence				
Urban	91 (90.1%)	36 (94.7%)	0.753	0.386
Suburban/rural	10 (9.9%)	2 (5.3%)		
Physical conditions				
Yes	38 (37.6%)	15 (39.5%)	0.040	0.841
No	63 (62.4%)	23 (60.5%)		
Preexisting mental disorders				
Yes	11 (10.9%)	1 (2.6%)	2.388	0.122
No	90 (89.1%)	37 (97.4%)		
Community-level COVID-19 contact				
Yes	23 (22.8%)	6 (15.8%)	0.815	0.6367
No	78 (77.2%)	32 (84.2%)		
[1]Time spent browsing information				
<1 h	25 (24.8%)	6 (15.8%)	1.600	0.659
1-3 h	52 (51.5%)	22 (57.9%)		
3-6 h	17 (16.8%)	8 (21.1%)		
>6 h	7 (6.9%)	2 (5.3%)		
Preference for the nature of information				
Primarily positive	50 (49.5%)	21 (55.3%)	0.366	0.545
Half positive/half negative or primarily negative	51 (50.5%)	17 (44.7%)		
Number of channels used to obtain information	2.81±1.222	3.05±1.451	0.982*	0.328
Reliability of the information obtained	2.00±0.941	2.20±1.024	1.076*	0.284

Notes:* t-value

Table S2. Comparisons of mental health status between caregivers of persons living with dementia and mild cognitive impairment

CI subgroups	No. (%)	Anxiety		χ^2	*p*-value	Depression		χ^2	*p*-value	Sleep problem		χ^2	*p*-value
		Yes (N=65)	No (N=74)			Yes (N=49)	No (N=90)			Yes (N=14)	No (N=125)		
Dementia	101 (72.67%)	48 (47.5%)	53 (52.5%)	0.086	0.769	38 (37.6%)	63 (62.4%)	0.911	0.340	11 (10.9%)	90 (89.1%)	0.274	0.601
MCI	38 (27.33%)	17 (44.7%)	21 (55.3%)			11 (28.9%)	27 (71.1%)			3 (7.9%)	35 (92.1%)		

Appendix. Six questions about the community-level COVID-19 contact.

- Did you have close contact with any individual with confirmed or suspected COVID-19?
- Was there anyone confirmed or suspected with COVID-19 in your community and neighborhood?
- How long on average did you spend on browsing information per day during the COVID-19 pandemic?
- What type of information (positive or negative) did you prefer to access about the COVID-19 during the COVID-19 outbreak?
- What channels did you primarily use to obtain information about the COVID-19 during the pandemic?
- How reliable and authoritative was the information about the COVID-19 you obtained?
